# Extended half-life factor IX prophylaxis up to every 21 days in hemophilia B: a longitudinal analysis of the efficacy and safety in selected adult patients

**DOI:** 10.1016/j.rpth.2023.100195

**Published:** 2023-05-25

**Authors:** Ingrid Pabinger, Toshko Lissitchkov, Asuza Nagao, Lynda Mae Lepatan, Yanyan Li, Wilfried Seifert, Maria Elisa Mancuso

**Affiliations:** 1 Clinical Division of Haematology and Haemostaseology, Medical Clinic I, Medical University Vienna, Vienna, Austria; 2 Department of Coagulation Disorders and Anemia, Specialized Hospital for Active Treatment Joan Pavel, Sofia, Bulgaria; 3 Department of Hematology, Ogikubo Hospital, Tokyo, Japan; 4 Perpetual Succour Hospital, Cebu, Philippines; 5 CSL Behring, King of Prussia, Pennsylvania, USA; 6 CSL Behring, Marburg, Germany; 7 Center for Thrombosis and Hemorrhagic Diseases, IRCCS Humanitas Research Hospital, Rozzano, Milan, Italy; 8 Humanitas University, Pieve Emanuele, Milan, Italy

**Keywords:** clinical trial, factor IX, hemophilia B, prophylaxis, treatment

## Abstract

**Background:**

Extended half-life factor IX (FIX) products have revolutionized prophylactic treatment for patients with hemophilia B as patients maintain protective FIX levels with minimal occurrence of spontaneous bleeding. rIX-FP is an extended half-life FIX product that allows prolonged dosing intervals.

**Objectives:**

To assess individualized and prolonged prophylactic dosing interval up to 21 days in adult patients (≥18 years) with hemophilia B in the rIX-FP clinical trial program.

**Methods:**

Patients who were included in the PROLONG-9FP phase III study or who received rIX-FP during surgery could continue into an extension study for long-term assessment. Patients began 7-day prophylaxis with rIX-FP, and after 6 months, they could extend dosing intervals to every 14 days. In the extension study, adult patients could switch to a 21-day regimen if well-controlled on a 14-day regimen.

**Results:**

Eleven patients transitioned from a 7-day prophylaxis regimen to a 14-day regimen and finally to a 21-day regimen, 5 of whom were treated on demand at enrollment. Patients who switched to the 21-day regimen had a median annualized spontaneous bleeding rate of 0.0 across all regimens. The median observed FIX activity remained >5 IU/dL until day 21 after a single 100-IU/kg dose of rIX-FP. After 6 months on the 21-day regimen, 2 patients switched back to a 14-day regimen. No inhibitors, anaphylactic reactions, or thromboembolic events occurred.

**Conclusion:**

Patients who are well controlled on a once-weekly regimen might extend their treatment interval to 14 days, and in adult patients, further extension to up to 21 days (100 IU/kg) may be considered.

## Essentials

• Extended half-life factor IX products enable prolonged individualized dosing intervals.
• Some patients with hemophilia B can extend rIX-FP dosing regimens from 7 days to 14 or 21 days.
• rIX-FP is well tolerated with low annualized bleeding rates.
• Long-term efficacy and safety of extended half-life rIX-FP are confirmed in adults.

## 1 Introduction

Prophylaxis with factor IX (FIX) replacement therapy is the recommended treatment for hemophilia B [1]. However, owing to the rarity of the disease, there is limited evidence around the use of long-term prophylaxis in these patients [1,2]. Patients with severe (FIX, <1%) or moderately severe (FIX, 1%-2%) hemophilia B display varied bleeding phenotypes. Spontaneous joint bleeds are the hallmark of severe hemophilia. These frequent joint bleeds can cause debilitating pain and arthropathy, significantly impacting the quality of life [3], and the subsequent chronic joint damage is one of its most serious complications [4]. Maintaining a high circulating FIX level is generally associated with reduced spontaneous bleeding and improved clinical outcomes [5,6], although the optimal level is likely to vary from patient to patient based on their joint status, age, lifestyle, bleeding phenotype, and other factors.

The half-life of FIX is 18 to 34 hours [7], meaning patients require regular intravenous infusions (2-3 times per week) with a standard half-life (SHL) recombinant FIX (rFIX) or plasma-derived FIX replacement therapy [8,9]. The burden of frequent infusions is known to be one of the main reasons for patients not adhering to their prophylaxis regimen [8,10]. The introduction of extended half-life (EHL) FIX replacement products has made it possible for patients to extend dosing intervals while maintaining higher FIX activity levels to minimize the occurrence and severity of bleeding [7], which is particularly important for those with complex disease. It is anticipated that extending dosing intervals with EHL products could provide a change in treatment behavior, leading to greater adherence to prophylaxis, and may enable patients to achieve optimal prophylaxis outcomes, such as low annualized bleeding rates (ABRs). EHL products can also be a treatment option, where appropriate, for patients who are treated on demand. Individualized and prolonged prophylaxis dosing regimens may improve adherence, as well as the quality of life, by reducing the burden of treatment associated with SHL products [9].

A recombinant fusion protein genetically linking human coagulation FIX with human albumin (rIX-FP) has been developed for use in patients with hemophilia B [11,12]. rIX-FP has shown an improved pharmacokinetic (PK) profile, with an approximately 5-fold longer half-life than that of SHL rFIX products [11,12]. The long-term safety and efficacy of rIX-FP have been demonstrated in a phase IIIb extension study in both adult/adolescent [13] and pediatric [14] previously treated patients (PTPs) with hemophilia B. Here, we report the longitudinal efficacy and safety of rIX-FP in patients whose prophylaxis treatment interval transitioned from 7 days to 14 days and up to 21 days during the PROLONG-9FP clinical trial program.

## 2 Methods

PTPs with hemophilia B (FIX, ≤2%) were enrolled in the PROLONG-9FP phase III study and were treated with rIX-FP between February 2012 and July 2014 [11,12]. Adult/adolescent and pediatric PTPs continued into the phase IIIb extension study and were treated with rIX-FP prophylaxis up to June 2018; patients could also enroll in the extension study following surgery with rIX-FP [13,14].

Treatment intervals during the program were determined by the investigator based on the dosing interval in the lead-in study and/or investigator and patient preference; at any 6-month treatment period during the study, all patients could remain on their treatment interval or change to an extended treatment regimen. At enrollment, patients were treated on-demand with rIX-FP or assigned to a 7-day prophylaxis regimen at 35–50 IU/kg rIX-FP as determined by the physician [12], and intervals could be extended to 10 or 14 days (50–75 IU/kg) if well-controlled on a 7-day regimen. Patients could continue into the extension study and were treated with their current regimen until a 6-month review period. During the extension study, those aged ≥18 years who were well controlled on a 14-day regimen for at least 6 months could extend their dosing interval to 21 days at a dose of 100 IU/kg. To be eligible to switch, patients must have completed ≥6 months of treatment with a stable dose on the 14-day prophylaxis regimen and have had no spontaneous bleeds for at least 2 months before switching. Here we analyze the treatment effects in patients aged ≥18 years who were initially treated on a 7-day prophylaxis regimen before treatment was extended to 14-day intervals and, for those who were well-controlled and fulfilled the eligibility criteria, further individualized with extended dosing intervals up to 21 days during the study.

Eligible PTPs, as determined by the investigator, and those who consented to receive 100-IU/kg rIX-FP every 21 days during the extension study underwent PK evaluation after a single 100-IU/kg rIX-FP infusion. The PK results were not used to determine eligibility to switch regimens. The efficacy and safety data of those patients who switched to the 21-day regimen were compared with their clinical outcomes reported during their treatment on the 7- or 14-day regimens. Dosing frequency, ABR, annualized spontaneous bleeding rates (AsBRs), factor consumption, and adverse events (AEs) were evaluated as previously reported [12]. ABR was estimated using the PTP’s observed data only if a PTP completed ≥12 weeks of treatment on the given regimen. Prophylaxis compliance was also measured, and it was defined as follows: (the number of on-schedule prophylaxis infusions)/(expected number of prophylaxis infusions) × 100, where on-schedule prophylaxis infusions are infusions during the study period that occur at the expected dosing frequency.

## 3 Results

Patient characteristics are described in Table 1: the mean (SD) age of these patients was 40.2 (12.79) years. Overall, 59 adult/adolescent PTPs enrolled in the extension study. This analysis reports data from a subset of 11 PTPs aged ≥18 years who transitioned from a 7-day prophylaxis regimen to a 14-day regimen and finally to a 21-day regimen.

**Table 1:** Characteristics of patients enrolled into the extension study who switched to the 21-day regimen.

Characteristics	N = 11
No. of patients	11
Age (y), mean (SD)	40.2 (12.79)
Weight (kg), mean (SD)	69.4 (9.84)
Race, n (%)	
White	7 (63.6)
Asian	4 (36.4)
Ethnicity, n (%)	
Not Hispanic or Latino	11 (100)
BMI (kg/m^2^), mean (SD)	22.7 (2.65)
BMI category, n (%)	
<30 kg/m^2^	11 (100)
≥30 kg/m^2^	0 (0)

BMI, body mass index.

A study flow diagram summarizing the different arms and regimens used by PTPs from the lead-in study and the final regimens at the end of the extension study is shown in the Figure. Of the patients treated at some point during the study with the 21-day prophylaxis regimen, 9 patients transferred from the main study and 2 patients enrolled directly in the extension study. Five of these 11 patients were originally treated on demand prior to enrollment in the PROLONG-9FP clinical trial program. Of these patients, only 1 showed evidence of arthropathy at enrollment. After 6 months on the 21-day regimen, 2 patients switched back to a 14-day regimen: 1 patient opted to switch to a more frequent regimen after 1 traumatic bleed and 1 patient switched due to the occurrence of breakthrough bleeding. The 2 patients who switched back to a 14-day regimen were not the patients who enrolled directly in the extension study.

**Figure:**
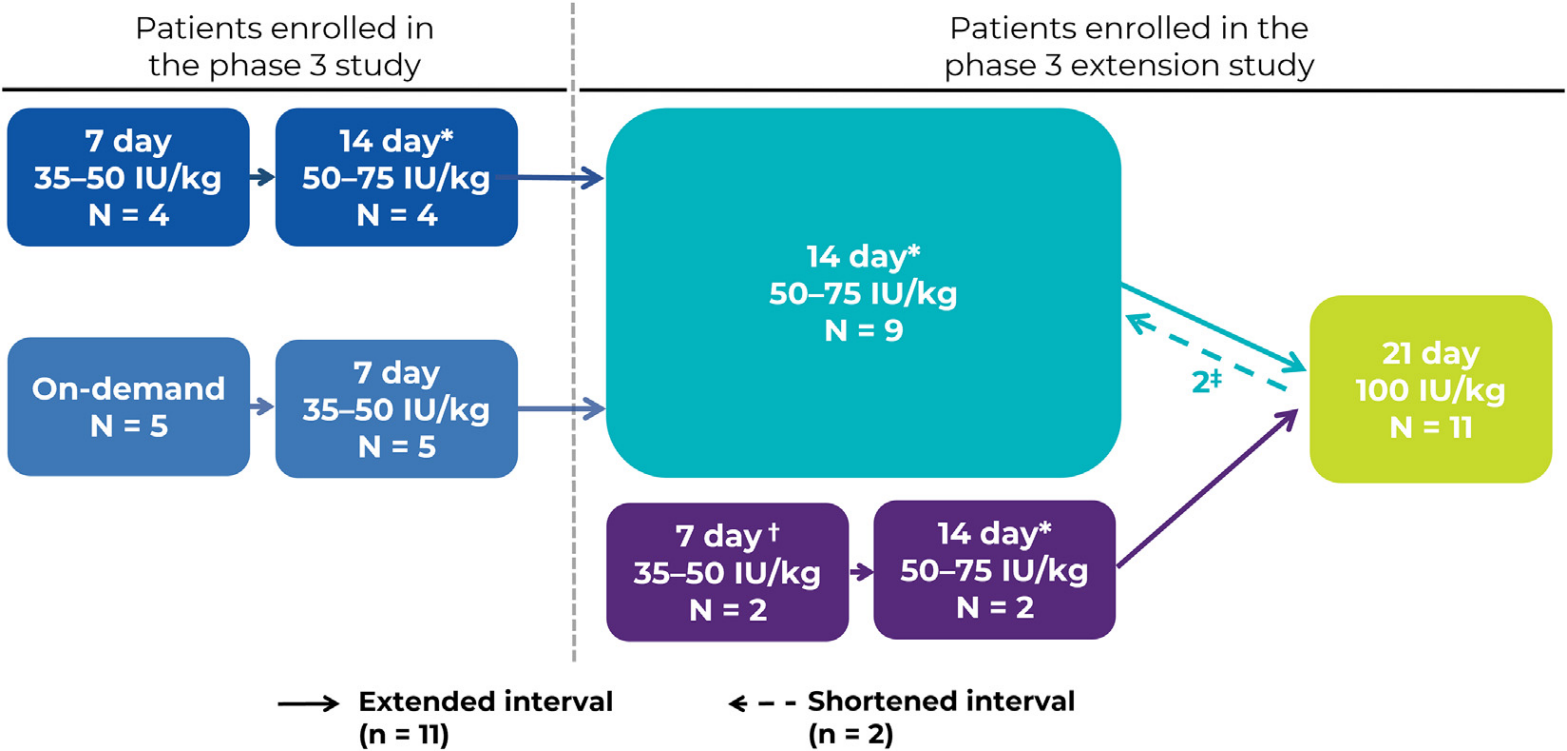
Change in regimens as patients moved through the clinical trial program to the 21-day prophylaxis dosing interval. ∗Prior to switching to the 14-day regimen, patients were required to have ≥6 months of rIX-FP treatment on their 7-day regimen, with no dose adjustments or spontaneous bleeding in the prior month. †Two patients were enrolled directly into the extension study following surgery with rIX-FP. ‡After 6 months of treatment on the 21-day regimen, 2 patients switched back to the 14-day regimen.

### 3.1 PK assessments

Before switching to a 21-day regimen, patients aged ≥18 years (n = 11), who were well controlled on the 14-day regimen for at least 6 months and therefore eligible to extend their dosing interval, underwent PK analysis following a single intravenous dose of 100-IU/kg rIX-FP; the PK parameters are reported in Table 2. One patient had 2 PK measurements available; when calculating the group means, the mean of these 2 measurements was taken. The median (range) steady-state trough level for the 21-day regimen was 7.6% (3.9-11.0 IU/dL). The median (range) steady-state trough levels from these patients treated on the 7- and 14-day regimens were 18.6% (12.6-32.4 IU/dL) and 13.9% (10.6-21.5 IU/dL), respectively. A post hoc listing showed that the PK parameters of the 9 patients who stayed on the 21-day regimen were not meaningfully different from those of the 2 patients who switched back to a more frequent regimen (data not shown).

**Table 2:** Pharmacokinetic parameters in patients who extended their treatment interval to a 21-day regimen.

Parameter	N^a^	PK value, mean (SD)
C_max_^b^ (IU/dL)	11	100.60 (12.69)
IR^b^ ([IU/dL]/[IU/kg])	11	1.00 (0.13)
t_1/2_ (h)	7	148.98 (19.62)
AUC_0-inf_ (h × IU/dL)	7	17 582.50 (2231.12)
CL (mL/h/kg)	7	0.59 (0.07)

AUC_0-inf_, area under the concentration time curve at time 0 extrapolated to infinity; CL, clearance; C_max_, maximum concentration; IR, incremental recovery; IU, international unit; PK, pharmacokinetic;t_1/2_, half-life.

a When calculating the group means, 1 patient had 2 pharmacokinetic data points available; in this instance, the mean of the 2 values was taken.

b Data are mean baseline–corrected for maximum concentration and incremental recovery, and all other parameters are baseline uncorrected.

### 3.2 Efficacy

Patients who switched to the 21-day regimen had a median AsBR of 0.0 on all prophylaxis regimens throughout the study (Table 3), and median ABR was similar across the 7-, 14-, and 21-day regimens. Of these patients, 64% (7 of 11) reported 0 spontaneous bleeds while on the 21-day regimen.

**Table 3:** Bleeding rates in patients switching to a 21-day regimen.

Regimen	On demand (N = 5)	7-day regimen (N = 11)	14-day regimen (N = 11)	21-day regimen (N = 11)
AsBR				
Mean (SD)	13.89 (7.86)	0.14 (0.477)	0.23 (0.596)	0.60 (1.408)
Median (range)	15.63 (11.7-16.7)	0.0 (0.0-1.6)	0.0 (0.0-2.0)	0.0 (0.0-4.7)
Patients with 0 spontaneous bleeds, n (%)	0 (0)	8 (73)	6 (55)	7 (64)
ABR				
Mean (SD)	16.17 (8.98)	0.52 (0.78)	0.44 (0.79)	1.19 (1.57)
Median (range)	17.58 (2.0-27.0)	0.00 (0.0-1.9)	0.00 (0.0-2.0)	0.32 (0.0-4.7)
Prophylaxis compliance (%)				
Mean (SD)	94.7 (10.65)^a^	95.0^b^ (6.32)	98.3 (3.79)	96.1 (5.61)

ABR, annualized bleed rate; AsBR, annualized spontaneous bleed rate.

a Compliance in the on-demand group refers to the 5 patients who were previously treated on demand but then switched to prophylaxis during the phase III study.

b Data based on 4 patients from the phase III study.

Both patients who enrolled directly from the phase III extension study following surgery with rIX-FP had a history of arthropathy. One patient, who had a history of arthropathy in both ankles, also experienced a bleeding event in the knee joint during the study, which was treated with a single dose of rIX-FP. Four out of the 5 patients who were treated on demand prior to enrollment had no target joints and experienced no joint bleeds during the 21-day regimen. One patient had arthropathy in both ankles and the right elbow, and experienced 2 bleeds in the left ankle and 2 bleeds in the left knee.

Patients could reduce their annual infusions by two-thirds from 52 infusions on a 7-day regimen to 18 infusions on a 21-day regimen. Overall, the majority of patients treated on the 21-day regimen were prophylaxis-compliant during the study, and prophylaxis compliance was similar (>94%) across all treatment regimens prior to switching to the 21-day regimen (Table 3). In those patients who were treated on a 21-day regimen, factor consumption reduced by approximately 18% compared with that on the 7-day regimen and by approximately 12% compared with that on the 14-day regimen (Table 4).

**Table 4:** Factor consumption across individualized treatment intervals.

Regimen	On demand (N = 5)	7-day regimen (N = 11)	14-day regimen (N = 11)	21-day regimen (N = 11)
Duration on regimen (d)				
Mean (SD)	187.0 (10.2)	308.1 (145.4)	516.2 (413.5)	654.5 (333.8)
Median (range)	185.0 (175.0-203.0)	231.0 (169.0-525.0)	323.0 (183.0-1361.0)	819.0 (147.0-1131.0)
No. of prophylaxis infusions annually				
Mean		52	26	17.5
Annualized FIX consumption (IU/kg)				
Mean (SD)		178.7 (24.63)	166.6 (6.21)	146.9 (5.53)

FIX, factor IX.

### 3.3 Safety

No inhibitors, anaphylactic reactions, or thromboembolic events were reported during the phase III or extension studies. During the phase III study, 9 of 11 (81.8%) patients reported 41 AEs, of which 37 were mild in severity and 4 were moderate in severity. During the extension study, 10 of 11 (90.9%) patients reported AEs, with 89.7% (26 of 29) considered mild and 6.9% (2 of 29) considered moderate in intensity; 1 severe AE (peritonsillar abscess) was reported 8 days after the last rIX-FP dose, and all AEs and the serious adverse event were unrelated to rIX-FP treatment. All of the AEs were resolved at the end of the study.

## 4 Discussion

To our knowledge, this is the first study to investigate extended dosing intervals of 21 days in patients with hemophilia B. This longitudinal analysis has demonstrated that a 21-day prophylaxis regimen with a rIX-FP dose of 100 IU/kg is an additional treatment option for selected adult patients (≥18 years) who are well controlled on a 14-day prophylaxis regimen. PTPs maintained low ABRs with rIX-FP throughout the clinical trial program and could reduce their infusion frequency without increasing factor consumption. Extending dosing intervals up to 21 days also provides an additional treatment option for patients who switch from on-demand treatment to prophylaxis. rIX-FP is the first factor replacement therapy indicated for dosing intervals longer than 14 days in adult patients in the European Union (EU) [15].

Among the 11 patients who were eligible to extend to the 21-day regimen, the AE profile was consistent throughout the trial regardless of their regimen. The safety profile of the 21-day regimen in patients ≥18 years was similar between the various prophylaxis regimens in the entire population across the clinical trial program [13]. With respect to safety and risks of the extended regimens, no clinical signs of thrombotic or thromboembolic complications were reported, which is consistent with the main study [13,14]. After administration of a single 100-IU/kg rIX-FP dose, median observed FIX activity remained >5 IU/dL until day 21, which corresponds to the FIX activity of a patient with moderate hemophilia [1]. The main objective of prophylaxis is to convert severe and phenotypically severe hemophilia into phenotypically moderate hemophilia to avoid spontaneous bleeding [1]. Study patients treated on a 21-day regimen also demonstrated reduced factor consumption with comparable ABRs to patients treated on a 7- or 14-day regimen, suggesting that efficacy is not compromised with extended dosing intervals.

PK parameters vary from patient to patient for several reasons [16]; this raises the possibility that the patients who were eligible to extend their dosing interval to 21 days may represent those with more favorable PK parameters compared to the patients who were not eligible. Although a direct comparison between patients who progressed to the 21-day regimen and those who did not is beyond the scope of this study, an indirect comparison between data from the phase I study demonstrated more favorable mean steady-state trough levels in patients who switched to the 21-day regimen. The median FIX trough levels for the 11 patients on the 21-day regimen presented here were 18.6%, 13.1%, and 7.6% for the 7-, 14-, and 21-day regimens, respectively, compared to the phase I study where the PK analysis of 50 IU/kg of rIX-FP demonstrated mean FIX levels of 13.41 IU/dL and 5.54 IU/dL for the 7- and 14-day regimens, respectively (*n* = 14) [9]. The concept of extravascular (EVS) distribution of FIX has been raised as a potential explanation for the difference in PK values between different FIX products [17,18]. Despite this, the relevance of FIX distribution in the EVS remains unclear and to date, no one has demonstrated the clinical relevance of this [17]. Furthermore, there are no studies looking at the biodistribution of FIX in the EVS in humans and its relationship to clinical outcomes remains purely hypothetical [17]. What has been demonstrated is that EHL products, including rIX-FP, provide effective bleed protection in patients with hemophilia B and protect/prevent the deterioration of joint health regardless of EVS distribution [11,13,14,[19], [20], [21], [22], [23], [24], [25], [26]].

There is evidence for the use of EHL-FIX products at less-frequent dosing regimens than SHL-FIX products while demonstrating maintained or improved long-term efficacy and safety. rIX-FP demonstrates favorable efficacy in terms of infusion frequency and clinical outcomes when indirectly compared with other EHL-FIX products. Patients receiving 100-IU/kg rFIXFc every 8 to 16 days (median prophylaxis interval, 13.7 days for adult/adolescent subjects) had a median ABR of 2.3 and median AsBR of 0.7 [20]. Although this is higher than that observed in this study with rIX-FP, target trough levels for rFIXFc were much lower than those achieved with rIX-FP (target FIX activity levels were 1%-3% in the rFIXFc trial vs 7.6% achieved with 21-day prophylaxis with rIX-FP) [13,21]. Long-term weekly administration of 40-IU/kg N9-GP achieved a median ABR of 1.03 and a median AsBR of 0.0 with mean steady-state FIX levels of 27.3% [19]. Dosing intervals longer than 7 days have not been evaluated using N9-GP, and the weekly regimen is the only one currently licensed. It should also be noted that in the EU, N9-GP is not currently indicated to treat children <12 years as its long-term safety has not yet been established.

In clinical practice, an individualized dosing approach takes into consideration all available information about the patient, including the bleeding phenotype (ie, age at first joint hemorrhage and frequency and severity of bleeding), the PK profile of the replacement factor, the individual patient’s level of physical activity and perceived risk of traumatic bleeding, the presence or absence of joint disease, and the patient’s adherence to the dosing regimen. Each patient’s information helps optimize prophylaxis regimens in clinical practice and can minimize the risk of joint bleeding. Although not all patients were suitable to switch to the extended 21-day dosing regimen, this study can be used as a baseline for tailoring the prophylaxis regimen to individual patient characteristics (individualized and flexible dosing) and adjusting them over time, as needed. This individualized dosing can improve outcomes and could potentially lead to cost savings in patients with hemophilia B [27,28]. Retrospective analyses of patient chart data have also demonstrated that patients who switch to rIX-FP can reduce their infusion frequency while maintaining low ABRs and reducing factor consumption [27,28].

Based on PK modeling simulations, the 21-day dosing regimen was investigated in patients aged ≥18 years only due to concerns over the faster clearance in younger patients and the potential pushback from regulatory authorities [11,12]. In July 2020, the European Medicines Agency approved the new Summary of Product Characteristics for rIX-FP, which now includes extended dosing options. The routine prophylaxis dosing regimen has been updated to include new data about the possibility of further extension of the treatment interval up to 21-day dosing for selected adult patients (aged ≥18 years).

This study was designed to evaluate the long-term effects of rIX-FP and data can be used to help guide clinical decisions; however, we acknowledge that there were some limitations with the study design. Patients were required to meet certain criteria to extend dosing to every 21 days; as such, only 11 patients in the extension study were treated with this regimen, suggesting that there may be limitations with data interpretation and that this regimen may only be suitable for a selected group of individuals. Patients aged <18 years were not eligible to receive the 21-day regimen, so the potential clinical benefits of extended dosing intervals in these patients could not be evaluated. While this long-term analysis demonstrates that switching to the EHL product rIX-FP may provide dosing flexibility of up to 21 days with prophylaxis in adult patients, which led to approval of further extension of the treatment interval of rIX-FP in the EU for some well-controlled patients aged ≥18 years on a 10- or 14-day dosing interval, other EHL rFIX products have not been evaluated in 21-day dosing intervals; thus, data from this article cannot be applied to other FIX products.

## 5 Conclusion

This study adds to the scientific evidence for the effective long-term use of EHL-FIX therapies and demonstrated that a 21-day prophylaxis regimen with 100-IU/kg rIX-FP is an additional treatment option with a good efficacy and safety profile for selected adult patients with hemophilia B. Extending dosing intervals to up to 21 days enables the individualization of dosing and frequency, which reduces injection frequency and FIX consumption, minimizing the burden of treatment while maintaining excellent prophylactic efficacy.
